# Acute Psychotic Episode Precipitated by Opioid Withdrawal in a Case of Bipolar I Disorder

**DOI:** 10.7759/cureus.45538

**Published:** 2023-09-19

**Authors:** Darshini B Shah, Palak A Fichadia, Freya H Shah, Shirish S Patel, Ivanshu N Jain

**Affiliations:** 1 Medicine, GCS Medical College, Hospital & Research Center, Ahmedabad, IND; 2 Medicine, Smt. Nathiba Hargovandas Lakhmichand (NHL) Municipal Medical College (MMC), Ahmedabad, IND; 3 Internal Medicine, Landmark Medical Center, Woonsocket, USA; 4 Internal Medicine, Smt. Nathiba Hargovandas Lakhmichand (NHL) Municipal Medical College (MMC), Ahmedabad, IND; 5 Psychiatry, Windsor University School of Medicine, Cayon, KNA; 6 Psychiatry, Terrell State Hospital, Terrell, USA

**Keywords:** bipolar personality disorder, suboxone, acute psychotic episode, opioid withdrawal, opioid addiction

## Abstract

Psychosis is a state of mind where an individual loses touch with reality and cannot differentiate between their perceptions and the real world. They experience one or more of the following: delusions, hallucinations, disorganized speech or catatonic behavior. While it can have a sporadic onset, drug-induced psychosis is also very common. When a person consuming large quantities of a particular drug, such as opioids, stops consuming the drug and enters the rehabilitation stage, this is a vulnerable time due to abrupt chemical changes. It can predispose the individual to psychosis due to withdrawal from the drug. Here, we present a 30-year-old Caucasian female who underwent rehabilitation and was treated successfully with buprenorphine/naloxone (Suboxone) for eight months. However, due to a comorbid psychiatric condition, mania, she was not able to adhere to her medication regimen, which led to an abrupt discontinuation of her maintenance medication, and this led to psychotic symptoms, including agitation, hallucinations, delusions, and bizarre behavior surrounding her family and individual health. Later, she restarted on buprenorphine/naloxone, which led to a gradual recovery and disappearance of her psychotic symptoms.

## Introduction

Opioid addiction is a global epidemic for the past 20 years [1], linked to many harmful health outcomes, including fatal overdoses, spread of infectious diseases, and unfavorable social outcomes, such as public disorder, crime, and increased healthcare expenses. Opioids have been found to have mood-enhancing properties, such as pleasure and reduced mood disturbance, and have been implicated in changing emotional states and altering emotional reactions [2].

Sedation, wooziness, nausea, vomiting, constipation, physical dependence, tolerance, and respiratory depression are typical adverse effects of opioid treatment. Clinical troubles about physical dependence and addiction may hinder appropriate prescribing, resulting in insufficient pain management. Delayed stomach emptying, hyperalgesia, immunologic and hormonal abnormalities, muscular rigidity, and myoclonus are examples of less prevalent adverse effects. Constipation and nausea are the side effects of opioid use that are most frequently reported. When these drugs are used in quantities more than prescribed/considered safe for usage, opioid overdose (OOD) can occur; symptoms of OOD include drowsiness, slow breathing, pinpoint pupils, cyanosis, loss of consciousness, and death [3]. Constipation, in particular, might be challenging to treat because tolerance to these two side effects seldom develops [4].

Individuals addicted to opioids face many challenges as they battle this disease. When opioids are abruptly stopped in a dependent patient, they frequently cause a severe withdrawal episode. Along with an opioid craving, some of these symptoms may last weeks or months after the last opioid usage. All addictions should be treated as chronic diseases because it has been shown to improve patient outcomes. It has been demonstrated that opioid maintenance therapy reduces patients' use of illegal opioids [4].

According to the *Diagnostic and Statistical Manual of Mental Disorders (DSM-5)* criteria, signs and symptoms of opioid withdrawal include lacrimation or rhinorrhea, piloerection "goose flesh," myalgia, diarrhea, nausea/vomiting, pupillary dilation, photophobia, insomnia, autonomic hyperactivity (tachypnoea, hyperreflexia, tachycardia, sweating, hypertension, and hyperthermia), and yawning [5].

The *United States Food and Drug Administration *has approved three medications that the *World Health Organization* has suggested for the treatment of opioid use disorder: opioid receptor full-agonist methadone, opioid receptor antagonist naltrexone, and opioid receptor partial-agonist buprenorphine, which is marketed as sublingual or buccal tablets or films, a skin patch (used to treat pain), and extended-release parenteral formulations (injection or implant) [6]. Out of the available options, our patient was treated with buprenorphine oral tablet 8 mg TID (three times a day).

Buprenorphine is a semi-synthetic opioid derived from thebaine, a naturally occurring alkaloid of the opium poppy, *Papaver somniferum*. It is a partial mu receptor agonist. However, it has been speculated that buprenorphine may also be helpful in the treatment of opioid dependence. Buprenorphine has certain potential pharmacologic benefits over methadone in treating opioid use disorder because of its partial agonist qualities, including reduced respiratory depression, drowsiness, withdrawal symptoms, the danger of toxicity at higher doses, and the risk of diversion [4,6].

Compared to heroin, morphine, and other common opioid analgesics, buprenorphine has a higher affinity for and binds to opioid receptors more tightly, making it a strongly binding partial agonist. As a result, the opioid agonist action abruptly decreases, precipitating the onset of opioid withdrawal symptoms. Compared to spontaneous withdrawal, which develops gradually after stopping agonists, precipitated withdrawal can be more severe, and delirium has rarely been seen [6]. Although it has been described, psychosis that develops after stopping buprenorphine or other opioids is uncommon [7].

## Case presentation

We present the case of a 30-year-old female of Caucasian descent who was brought to Terrell State Hospital, Texas, USA, by her family with the presentation of bizarre behavior, delusions surrounding her family, and reproductive history, in addition to poor insights and agitation. Previously, she was diagnosed with bipolar I disorder and grand mal seizures, managed appropriately by levetiracetam 1000 mg BID (two times a day), sertraline 100 mg OD (once daily), a mood stabilizer (patient and family were unable to recall the name) and was taking hydroxyzine 50 mg BID for anxiety.

The patient had three episodes of grand mal seizures five days back and was taken to the Christus Mother Frances Hospital by her husband, where they increased the dosage of levetiracetam. Since her return from the hospital, her husband and mother noticed several changes in her behaviour. She was described as acting bizarrely, talking to herself, with incomprehensible speech, running outside, and screaming that she had a miscarriage, that her mother was dead, and that her dead brother was trying to rape her. She was brought to Terrell State Hospital the following day.

According to the patient, she had been under the influence of opioids (especially hydrocodone) for the past 11 years. Based on collateral information, she had recently stopped using opioids and had been sober for the past eight months. During this time, she was on buprenorphine/naloxone, with a dose of 8 mg/2 mg (3.5 tablets per day), with doubtful compliance.

On further reviewing her charts, she has a history of a suicidal attempt and was abused sexually by an outsider when she was young. She knew her assailant was in detention and scheduled to be freed next month, but she did not feel intimidated. She also stated that the experience did not affect her until her daughter turned three, which triggered her manic episode previously.

The goal of the treatment was to allow mood regulation and seizure control and to stabilize the psychotic symptoms. Therefore, she was treated with divalproex sodium 500 mg BID to stabilize mood and to control her seizure frequency, risperidone 2 mg BID for her psychotic symptoms, and benztropine mesylate 1 mg BID for extrapyramidal side effect (EPS) prophylaxis. In addition, she was started back on buprenorphine/naloxone 4 mg PO BID initially and then titrated up to 8 mg PO TID (as it was unclear when the patient had stopped taking buprenorphine/naloxone).

In addition, blood analysis was performed, and the results were mostly normal with the exception of slightly raised alkaline phosphatase (ALP) (105 U/L) and aspartate transaminase (AST) (59 U/L). To rule out organic reasons of psychosis, a neurologist's consultation was obtained; after that, a magnetic resonance imaging (MRI), computerized tomography (CT) scan, and electroencephalogram (EEG) were carried out, and the results were non-contributory. Hence, the patient did not seem to have an organic cause for her current presentation.

Risperidone was switched to olanzapine and was titrated up to 10 mg PO TID, chlorpromazine 200 mg PO TID as needed for aggression during her stay, benztropine mesylate 1 mg PO BID for EPS. Later on, chlorpromazine and benztropine mesylate were discontinued to minimize polypharmacy and cholinergic side effects.

Besides these medications, the patient continues to feel restless and experience body aches and flu-like symptoms. These symptoms correlate with opioid withdrawal. Based on this hypothesis, the dose of buprenorphine was titrated to 8 mg PO TID, as the patient requested once-a-day dosing. Hence, buprenorphine was increased to 24 mg PO QAM (every morning), which she tolerated well. Her symptoms improved significantly, and psychosis started to clear up. She appeared more awake and oriented, but there were still lingering symptoms of delirium. AST and ammonia were on an upward trend, so divalproex sodium was discontinued, and she was switched to lithium extended release, which was titrated up to 600 mg PO QHS (at bedtime). She tolerated the change well and was much better oriented, and her mood and psychosis improved significantly with the change. Olanzapine was also lowered to 10 mg PO BID.

The patient showed gradual improvement. On the day of discharge, she denied any visual/auditory hallucinations, depression, anxiety, or any other mood symptoms. She was able to answer all orientation questions appropriately. Her mood was euthymic, and the effect was mood congruent. Her speech became frequent every day with a regular tone and rhythm. No abnormal motor activity was noted. She denied any suicidal or homicidal ideations; in addition, her insights and judgment were excellent. The patient was discharged on the following medications: lithium 600 mg PO QHS, olanzapine 10 mg PO BID, buprenorphine/naloxone 24 mg/6 mg QAM, and levetiracetam 1000 mg PO BID. She was discharged to go back home to her family.

## Discussion

In this case report of a 30-year-old Caucasian female, we would like to discuss the pathogenesis of the sudden appearance of psychotic symptoms in the context of discontinuation of buprenorphine/naloxone.

Acute psychosis is a group of mental health symptoms where there has been a disconnect from reality. Some contributing factors for this condition could be genetics, substance abuse, trauma, psychiatric disorders (schizophrenia spectrum disorder, brief psychotic disorder, and delusional disorder), and medical conditions (e.g., infections in the central nervous system, lupus, and Lyme disease). It is well recognized that dysfunction in dopaminergic and glutamate pathways can lead to a psychotic episode. Dysregulation of inhibitory neurons can also precipitate the episode.

There are various neurological pathways and theories for the onset of psychosis, such as the disorder of connectivity and inefficient neural system stabilization. We centered our attention on the connection of the mesostriatal contacts, which have recently been strongly linked to psychosis. The aberrant striatal connection has received extensive study and documentation. According to recent findings, the dorsal (associative) striatum is the region where dopaminergic dysfunction is most frequently observed. As dopaminergic activity associated with enhanced dopamine synthesis and release in psychotic illnesses originates in the midbrain, the midbrain connection is of particular relevance [8].

Some cognitive models of trauma and post-traumatic stress disorder (PTSD) also show how intense or adverse experiences can lead to acute dissociation states. According to current theories, this reaction involves a desynchronization or dissociation of processing between the intellectual and perceptual components of the event, leaving unprocessed perceptual memories devoid of spatial, temporal, and conceptual contexts. Unusual perceptions in psychosis, such as hearing voices, may share phenomenological similarities with perceptual intrusions in PTSD, including flashbacks, which are disconnected and decontextualized perceptual memories [9]. Based on this study, it can be understood that the knowledge of her attacker being released from prison might have triggered a memory and contributed to her psychotic symptoms. However, since her hallucinations are vague and have no connection to the assailant, PTSD seems less likely to be the reason.

In addition, as our patient had increased AST and ALP, it is possible that she had some form of liver dysfunction, which could have led to her current presentation. A catecholamine that is produced as a byproduct of the dopamine biosynthetic pathway is norepinephrine (NE). NE levels are decreased by hepatectomy, liver devascularization, and thioacetamide-induced hepatic dysfunction in rats. In addition, NE-binding sites alpha _(α1) _and beta _(β1) _of the frontal cortex and thalamus have lower densities and are related to greater extracellular NE in hepatic coma. In the cerebral cortex of the portocaval shunted rat, norepinephrinergic receptors alpha _(α1 and α2)_ are also overexpressed. These findings suggest that NE may play a role in the pathogenesis of several neuropsychiatric diseases seen in hepatic encephalopathy (HE) [10]. However, HE has several additional signs and symptoms before manifesting psychosis, which were not seen in this patient. 

The patient had a history of bipolar I disorder, which can also present with psychotic features. However, the patient's mania was appropriately treated with mood stabilizers, as evidenced by her medical history. The following pathophysiology describes how opioids interact with a variety of receptors and neurological pathways to alter our central nervous system: Any opioid, including heroin, oxycodone, and others, binds to mu-opioid receptors on the surfaces of opioid-sensitive neurons as it circulates through the circulation to the brain. When these chemicals bind with the receptors, the exact biochemical brain mechanisms that make people feel good after engaging in activities, such as eating and having sex, are triggered. Opioids are prescribed therapeutically to treat pain, but when they trigger these reward mechanisms when there is not much pain present, they can promote recurrent drug use just for pleasure [11].

Opioids affect several brain circuits, including the mesolimbic (midbrain) reward system. The nucleus accumbens (NAc) releases the chemical dopamine (DA) because of signals produced by this system in the ventral tegmental area, a region of the brain. This DA release into the NAc brings on feelings of pleasure. Other parts of the brain produce a permanent record or memory that links these positive emotions with the events and surroundings in which they take place. When a person with substance use disorder encounters those people, places, or things again, these memories, known as conditioned associations, frequently cause a yearning for drugs, which motivates the patient to look for more drugs despite several barriers [11].

According to a few studies, opioid withdrawal presenting as an acute psychotic episode is very rare but has been seen with synthetic opioids, such as tramadol, oxycodone, and buprenorphine, in some clinical scenarios [12,13].

Buprenorphine is a partial mu-opioid receptor agonist and kappa-opioid receptor antagonist. There are two mechanisms by which buprenorphine withdrawal precipitates psychosis: Partial agonism on serotonin 2A receptor (5-HT_2A_) causes an indirect effect on glutamate release in the prefrontal cortex and putamen, and it also has a moderate antipsychotic effect due to kappa antagonism. Hence, withdrawal can unmask psychosis [14].

In a few case reports, buprenorphine withdrawal presented with hallucinations, paranoid delusions, agitation, aggression, and suicidal thoughts (both typical and atypical symptoms of psychosis) [7,15]. These symptoms were only partially improved by adding antipsychotics. There was a complete remission of symptoms only after reintroducing buprenorphine. This study resembles the progression of symptoms and remission of the same in our patient. A similar study showed complete improvement in psychosis after adding buprenorphine, even after a few weeks, which suggests the importance of a thorough psychiatric history to rule out substance abuse, comorbidities, and family history. It will help prevent the development of acute psychotic episodes [7,12]. 

Hence, based on previous studies and the presentation of our patient, we can safely postulate that buprenorphine withdrawal can precipitate psychosis, and it can be treated mainly by adding a combination of antipsychotics and buprenorphine.

## Conclusions

Based on the patient's history of opioid use disorder and current treatment with buprenorphine/naloxone, it is possible to hypothesize that her recent episode of psychosis was due to a sudden withdrawal from buprenorphine/naloxone (mainly due to its buprenorphine component) rather than a manifestation of her mania. Because buprenorphine works on the same neurological pathways as psychosis, her withdrawal from buprenorphine/naloxone and precipitation of psychosis overlap in this context and are likely to have followed each other. Our study, however, is limited to a single case presentation, and we would suggest conducting further studies to investigate the hypothesis at a more detailed and nuanced level.
